# Unfolded protein response plays a critical role in heart damage after myocardial ischemia/reperfusion in rats

**DOI:** 10.1371/journal.pone.0179042

**Published:** 2017-06-07

**Authors:** Chengcheng Zhang, Yi Tang, Yanming Li, Liang Xie, Wei Zhuang, Jing Liu, Jianbin Gong

**Affiliations:** Department of Cardiology, Jinling Hospital, Nanjing University School of Medicine, Nanjing, Jiangsu, China; Duke University School of Medicine, UNITED STATES

## Abstract

The unfolded protein response (UPR) plays a critical role in cell death mediated by ischemia/reperfusion (I/R) injury. However, little is known about the exact mechanism of UPR signaling pathways after myocardial I/R injury in rats. An attempt was therefore made to assess whether the myocardial I/R induced UPR, and which branch of UPR (ATF6, IRE1 and PERK) signal pathway was activated. Sprague-Dawley rats were pretreated with UPR stimulator dithiothreitol (DTT) and UPR inhibitor 4-phenylbutyrate (4PBA) and then subjected to myocardial I/R surgery. Compared with sham-operated group, the expression of GRP78, ATF6, CHOP and sXBP1 in the I/R injured group is significantly increased at transcript and protein levels, which indicated that all the three signal pathways of UPR were activated in the myocardial I/R injury. Compared with the I/R injured group, treatment with 4PBA effectively decreased myocardium infarct size, reduced myocardial apoptosis, down-regulated caspase-12 expression, diminished serum creatine kinase and lactate dehydrogenase levels. In contrast, these effects were reversed in DTT treated group. In summary, these results demonstrated that myocardial I/R injury activates UPR and inhibiting cell UPR possesses a cardioprotective effect through the suppression of ER stress-induced apoptosis. Therefore, inhibition of UPR might be used as a therapeutic target during myocardial I/R injury.

## Introduction

Ischemic heart disease including acute coronary syndrome remains the leading cause of mortality and disability worldwide [1]. The most efficient treatment of ischemic cardiovascular disease is timely reperfusion, including primary percutaneous coronary intervention and thrombolytic therapy. However, reperfusion itself results in a further cardiomyocyte damage, which is commonly referred to myocardial ischemia/reperfusion (I/R) injury [2, 3]. It has been shown that I/R injury triggers many distinct and overlapping cell signaling pathways, and finally decide the cell to survival or death [4].

The endoplasmic reticulum (ER) is a multifunctional cellular organelle, which is recognized as the principal site of protein biosynthesis and folding, lipid biosynthesis, cell homeostasis, calcium homeostasis and apoptosis [5]. Under cellular stress conditions, such as ischemia, hypoxia, depletion of ER Ca^2+^ stores, exposure to free radicals, and accumulation of unfolded/misfolded proteins, disrupt the proper function of ER, all of which can potentially induce ER dysfunction, collectively known as ER stress [6–10]. When ER stress was generated and sustained, then the unfolded protein response (UPR) was activated to deal with this disadvantageous situation [11]. Actually, UPR acts as a sentinel of protein folding in the ER, restoring the ER homeostasis, while the cell death or apoptosis will ensue with the UPR continuing [12]. In the eukaryotic cells, UPR is characterized by the activation of three ER transmembrane effector proteins: transcription factor-6 (ATF-6), inositol requiring enzyme 1 (IRE1), and PKR-like ER kinase (PERK) [13], which mediated three branches signal pathway of UPR. Glucose-regulated protein 78 (GRP78), is one of the best characterized UPR target genes in ER. Upon ER stress, sequestration of GRP78 with unfolded proteins will activate the three UPR sensors [14]. Activated IRE1 pathway splices the mRNA of a transcription factor called X-box-binding protein-1 (XBP1), removing a 26-bp nucleotide intron from the full-length XBP1 mRNA that creates a translational frame shift leading to the expression of a spliced XBP1 (sXPB1) [15, 16]. Spliced XBP1 is a highly active transcription factor for ER-resident enzymes and chaperones [15]. Activated PERK phosphorylates eukaryotic translation initiation factor 2 subunit α (eIF2α), which leads to inhibition of global protein synthesis [17]. However, phosphorylated eIF2α can also lead to an increase expression of ATF4 and CHOP [18]. When ER stress is excessive and/or prolonged, however, apoptotic signals are initiated by the UPR in the ER, including induction of C/EBP homologous protein (CHOP), activation of Jun N-terminal kinase (JNK), and cleavage of caspase-12 [19–21].

Myocardial I/R injury negatively regulates protein synthesis, leading to the activation of numerous signaling pathways from the ER to the cytosol and nucleus, which mainly represent UPR and ER-associated protein degradation (ERAD) [22]. Previous studies demonstrated that during I/R injury in the myocardium ATF6 branch of UPR is activated and help to reduce the I/R injury [23, 24]. The protein CHOP and caspase-12 were involved in ER stress-induced apoptosis, which were confirmed in many studies [25–27]. However, to the best of our knowledge, whether myocardial I/R injury activate the three branches of UPR pathway is still largely unknown.

In this study, we pretreated with UPR stimulator dithiothreitol (DTT) and UPR inhibitor 4-phenylbutyrate (4PBA) on rat myocardial I/R, and examined which UPR pathway is involved in the myocardial I/R injury. And we find that I/R injury activate all the three branches of UPR pathway. Moreover, inhibiting cell UPR possesses a cardioprotective effect via suppressing ER stress-induced apoptosis.

## Materials and methods

### Antibodies and reagents

Evans blue dye, 2,3,5-triphenyltetrazolium chloride, Dithiothreitol (DTT) and 4-phenylbutyrate (4PBA) obtained from sigma Aldrich, USA. Antibodies for GRP78, CHOP, ATF6, XBP1 and GAPDH were obtained from Santa Cruz Biotechnology, USA; Antibody for caspase-12 was obtained from Sigma Aldrich, USA. The secondary antibody HRP-labeled Goat Anti-Mouse IgG and Goat Anti-Rabbit IgG were obtained from beyotime, China.

### Creation of myocardial I/R rat model in vivo

Adult male Sprague-Dawley (SD) rats (250-300g) were procured from the Animal Center of Jinling Hospital (Nanjing, China). Animal experiments were conducted after approval and in accordance with the strict guidelines of the Institutional Animal Ethics Committee (IAEC). SD rats were pretreated with UPR stimulator dithiothreitol (DTT) before ischemia 10 minutes and UPR inhibitor 4-phenylbutyrate (4PBA) before ischemia 60 minutes and then subjected to myocardial I/R surgery. There have some study report that dithiothreitol (DTT) is a reducing agent and strongly induces ER stress [28]. Sodium 4-phenylbutyrate (PBA) is a low molecular weight fatty acid that acts as a chemical chaperone reducing the load of mutant or unfolded proteins retained in the ER during cellular stress and also exerting anti-inflammatory activity [29]. The rats were anaesthetized using pentobarbital sodium (3%) via intraperitoneal injection. The surgical procedures were performed as previously described [30]. Briefly, after pericardiotomy, a 6–0 silk suture was placed under the left coronary artery (LCA), which was occluded by tightening the snare using a lightweight hemostatic clamp. After 30 min of myocardial ischemia, the suture was loosened and the myocardium was reperfused for 6 hr (the myocardium of left ventricular for analysis the transcript and protein levels and the full left ventricular for quantification of myocardial apoptosis and infarct size), for 72 hr (for cardiac function determination). The sham-operated animals underwent the same surgical procedures except that the suture around the LCA was not fastened. Ischemia was confirmed by a transient decrease in blood pressure and cyanosis on the myocardium surface. Reperfusion was indicated by an epicardial hyperemic response and the rapid disappearance of cyanosis.

### Identification of myocardium infarct size in rats with I/R

At the corresponding time of the experiment, the rats were anaesthetized and the LCA was reoccluded, injected with 2% Evans blue dye via the external jugular vein, then the area at risk (AAR) in the heart can be displayed. About 5 min later, the heart was rapidly excised and frozen at -80℃ approximately 10 min. After that, the frozen heart was cut into 5 transverse slices of equal thickness. Then the slices were incubated in phosphate-buffered 1% 2,3,5-triphenyltetrazolium chloride (TTC) for 10 min at 37°C. In the end, the slices fixed with 4% paraformaldehyde prepared in PBS (pH-7.4). In the AAR area, where not stained by TTC (white) were confirmed as the area of infarction (AI). The AAR, AI and ventricle size (VS) were assessed by using Image-J software [31]. The AI and AAR were expressed as percentages of AAR and LV area respectively (AI/AAR and AAR/LV).

### Measurement of LDH and CK activities in rats with I/R

After the end of the reperfusion, plasma samples (1 mL) were obtained from carotid aorta using a heparinized syringe and immediately centrifuged. The activities of lactate dehydrogenase (LDH) and creatine kinase MB (CK-MB) were assayed using kits, which purchased from the Institute of Jiancheng Bioengineering (Nanjing, China).

### Apoptotic cell assay

Apoptosis was assessed by Annexin V-FITC and PI staining followed by analysis with flow cytometry. The methodology followed the procedures as described in the kit. Briefly, the tissue cells were collected by digestion of pancreatic enzyme, which has no EDTA. Then wash cells three times with cold PBS. Resuspended cells in 1 × binding buffer at a concentration of 1×10^6^ cells/mL and transfer 100 μL (1×10^5^ cells) to a 5 mL culture tube. Then added 5 μL of Annexin V-FITC and 10 μL of PI. Gently vortex the tube and incubate for 15 minutes at room temperature in the dark. Later on, 400 μL of 1 × binding buffer was added to each tube. The stained cells were analyzed by flow cytometry as soon as possible (within an hour).

For detection of DNA fragmentation, terminal deoxynu-cleotidyl transferase (TdT) -mediated dUTP nick end labeling (TUNEL) assay was performed as described previously [32]. Briefly, after in vivo I/R procedure, the myocardium of left ventricular was fixed in 10% buffered formalin and subsequently embedded in paraffin to obtain 5μm-thick sections. DeadEnd™ Fluorometric TUNEL system (Promega) was used to stain for apoptotic nuclei. The numbers of TUNEL-positive cells were expressed as a percentage of total cells. TUNEL-positive nuclei were counted by randomly selecting 10 fields of the mid ventricular section. Nuclei were stained by 4’, 6’-diamino-2-phenylen-dolehydrochloride (DAPI) (Invitrogen, USA) and then visualized under fluorescence microscope (Leica DM5000 B, Leica Microsystems). Six specimens were employed for apoptotic index analysis. The apoptotic index was calculated as the ratio of the number of TUNEL-positive neurons to the total number of nuclei.

### Echocardiography

Six rats from each group were used for cardiac function determination by Doppler echocardiography with a 15 MHz linear transducer (Visual Sonics Vevo 2100, Canada). To avoid interference in the acoustic signal by residual air trapped inside the chest cavity, echocardiography was conducted after 72 h of reperfusion, by which time most of the residual air had been absorbed. Baseline echocardiography was obtained 30 min before surgery. M-mode echocardiography was used to evaluate the cardiac dimensions and function. Left ventricular ejection fraction (LVEF) and left ventricular fractional shortening (LVFS) were calculated with computerized algorithms.

### Reverse transcription-polymerase chain reaction (RT-PCR)

For X-box binding protein (XBP1) analysis, cDNA was synthesized as previously mentioned. PCR was performed using Geneamp PCR system (Sigma) with Taq DNA polymerase (CWBIO, China). The primer specific for target sequences of XBP1: forward, GAGCAGCAAGTGGT GGAT; reverse, AGGCAACAGCGTCAGAAT. Amplification conditions included: 94℃ for 5 min; 30 cycles at 94℃ for 30 s, 58℃ for 30 s and 72℃ for 45 s; 72℃ for 5 min. When UPR is induced, XBP1mRNA is spliced and activated through activation of the ER stress sensor IRE1 [33]. The primer sets for XBP1 were amplified the PCR products of 297 bp for the unspliced form and 271 bp for the spliced form. The amplified RT-PCR products were separated by 3% agarose gel electrophoresis and visualized with ethidium bromide staining under UV light.

### Quantitative real-time PCR (qRT-PCR)

At the indicated times after reperfusion, hearts were removed and frozen in liquid nitrogen immediately then stored at -80℃. Total RNA was isolated from hearts using TRIZOL reagent (Invitrogen) according to manufacturer’s protocol. Complementary DNA (cDNA) was synthesized using the Primescript^TM^ RT reagent kit (Takara Bio, Dalian, China) according to the manufacturer’s instructions. Three-step quantitative real-time PCR was performed using the Thermal Cycler Dice^R^ Real Time System with SYBR ExScriptTM RT-PCR Kit (Takara Bio, Dalian, China). The primers used for target sequences of GRP78, ATF6, CHOP, Caspase-12 and GAPDH as following: GRP78: forward, ACTGGAATC CCTCCTGCTC; reverse, CAAACTTCTCGGCGTCAT; ATF6: forward, GCAGGTGTATTACGCTTCG; reverse, TTCGGTCTTGTGGTCTTGTT; CHOP: forward, CACAAGCACCTCCCAAAG; reverse, CCTGCTCCTTCTCCTTCAT; Caspase-12, forward: GGTCTTTATGTC CCACG; reverse, CAGTATGTCTGCCTCTGC; GAPDH: forward, GCA AGTTCAACGGCACAG; reverse, GCCAGTAGACTCCACGACAT. The amplification conditions were performed at 95°C for 10 minutes, followed by 40 cycles at 95℃for 10 seconds, 58℃ for 15 seconds and 72°C for 15 seconds. Levels of target gene mRNA were normalized to those of reference genes GAPDH.

### Western blotting analysis

Hearts were homogenized in five volumes of ice cold lysis buffer (200 mmol/l HEPES (pH 7.4), 250 mmol/l sucrose, 1 mmol/l dithiothreitol, 1.5 mmol/l MgCl_2_, 10 mmol/l KCl, 1 mmol/l EDTA, 1 mmol/l EGTA, 0.1 mmol/l PMSF, and 1% protease and phosphatase inhibitor cocktails (Sigma)). The lysates homogenates were spun at 12,000g for 5 min and the supernatant was used. For SDS-PAGE analysis an equal amount of protein (50 μg) was loaded in each well and subjected to 10% sodium dodecyl sulfate-polyacrylamide gel electrophoresis. The separated proteins were then transferred onto PVDF membrane (Millipore, MA, USA) and blocked with 5% non-fat dry milk prepared in 1×TBS. The membranes were incubated with the primary antibodies for 4 h at RT or overnight at 4℃ (dilutions 1:500–1:1,000). The following primary antibodies were used: GRP78, ATF6, CHOP, XBP1 and Caspase12. After washing the membranes were incubated with secondary antibodies (1:5000) for 2 h at RT. The secondary antibodies used were horseradish peroxidase (HRP) conjugated anti-IgG corresponding to the primary antibodies. The blots were developed by using ECL Plus detection system (Amersham Pharmacis). The relative band intensity was measured by Image J software.

### Ethics statement

Animal maintenance and experimental procedures were conducted after approved by the Animal Care Committee of Nanjing University (Nanjing, China) and in accordance with the strict guidelines of the Institutional Animal Ethics Committee (IAEC).

### Statistical analysis

All data shown in this study are presented as the mean ± SEM of three or more independent experiments. The statistical comparisons were performed using a 2-tailed Student’s t-test or the ANOVA test. * P<0.05 and ** P<0.01 were considered statistically significant.

## Results

### Suppression of UPR reduced myocardial infarct size after I/R in rats

**Fig 1:**
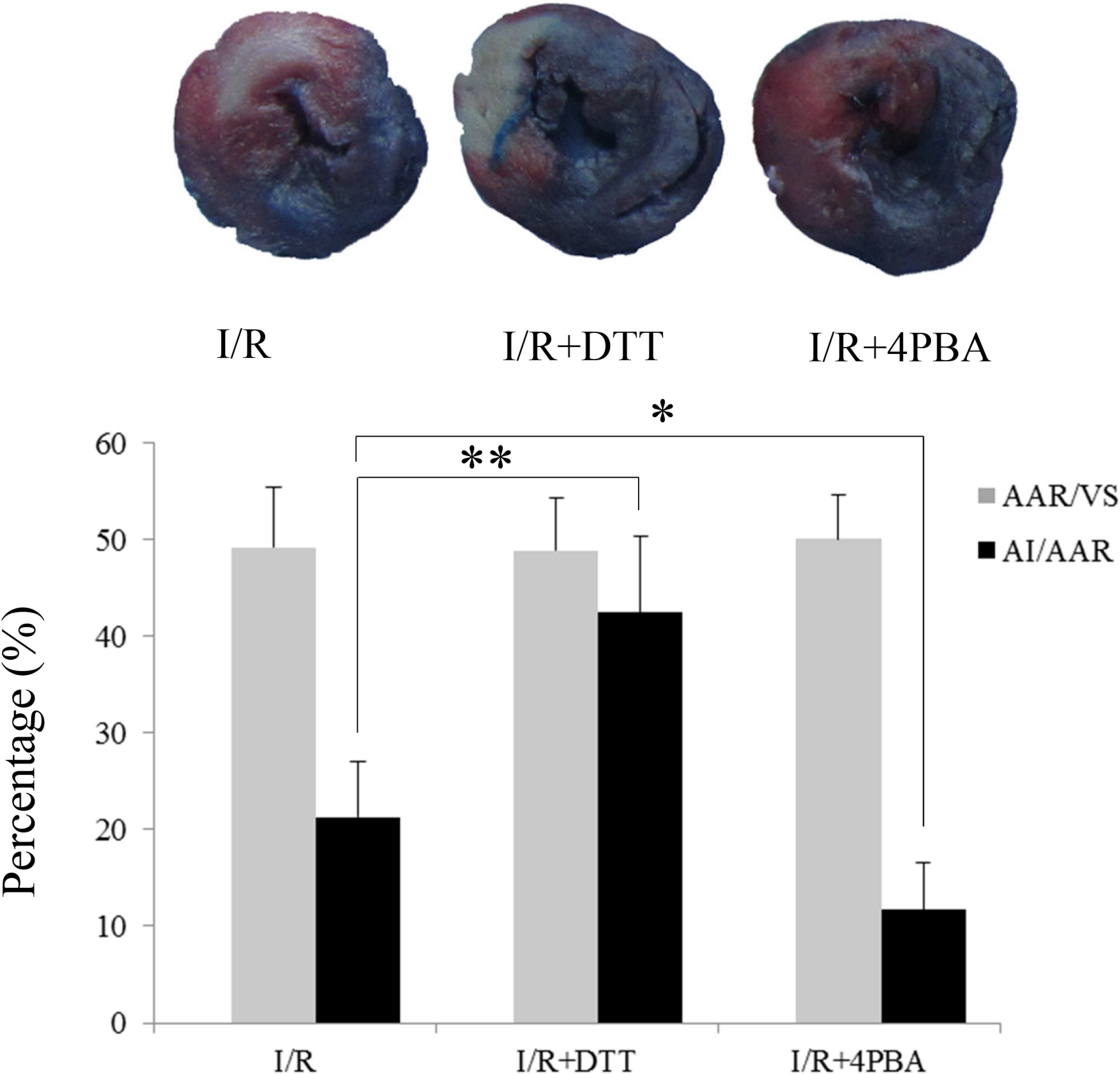
Effect of DTT and 4PBA preconditioning on myocardial injury in vivo. Effect of pretreat with UPR stimulator DTT and UPR inhibitor 4PBA on myocardium infarct size. The data were presented as the mean ± SEM (n = 6, *P<0.05, **P<0.01).

We utilized the infarct size using the Evans-TTC method to evaluate the effect of UPR on myocardial I/R injury [31]. In this study, the ratio of AAR to VS (AAR/VS) was used to express the area at risk, and the ratio of AI to AAR (AI/AAR) was used to express the area of infarct size. There were no differences in AAR/VS between the I/R, I/R+DTT, and I/R+4PBA groups. In rats with I/R group, the percentage of AI/AAR was approximately 21.32%. Compared to the I/R group, the I/R+4PBA group showed a significant reduction in myocardium infarct size by 11.67%. However, pretreatment with UPR stimulator DTT, the I/R+DTT group showed the percentage of AI/AAR was increased nearly by 42.48% (Fig 1).

### Suppression UPR reduced plasma LDH and CK-MB activities after I/R in rats

**Fig 2:**
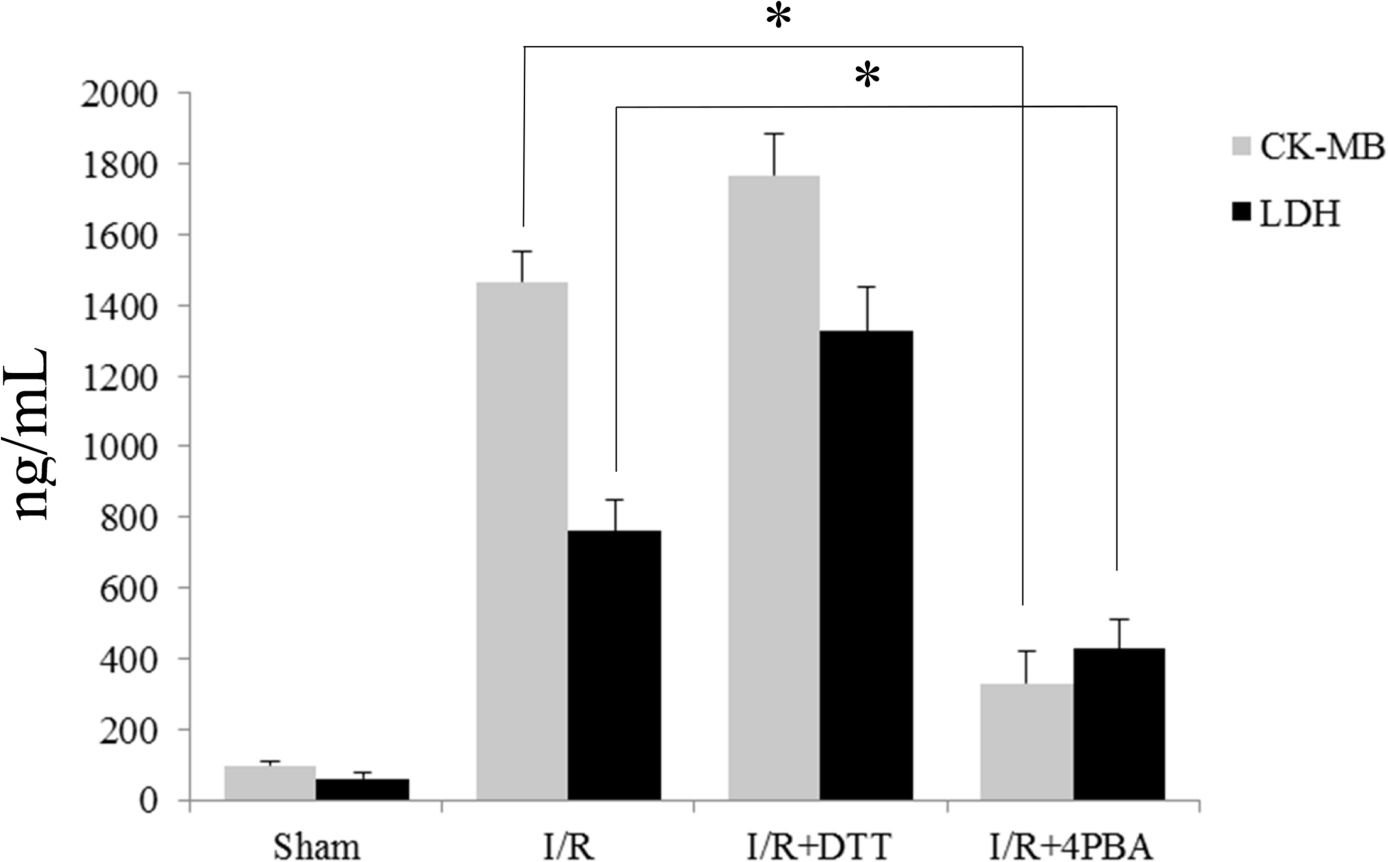
Effect of pretreat with UPR stimulator DTT and UPR inhibitor 4PBA on plasma lactate dehydrogenase (LDH) and creatine kinase MB (CK-MB) activities. The data were presented as the mean ± SEM (n = 6, *P<0.05).

In plasma, LDH and CK-MB activities can be used to evaluate the extent of myocardium injury. Compared to the sham group, the plasma LDH and CK-MB activities in the I/R group were significantly increased by 13 times and 15 times, respectively (*P*<0.01; Fig 2). Pretreatment with 4PBA significantly reduced plasma LDH and CK-MB content by 43.77% and 77.54%, respectively (*P*<0.01; Fig 2). However, pretreatment with UPR stimulator DTT, the plasma LDH and CK-MB content were higher than those in the I/R group (*P*<0.05; Fig 2).

### Myocardial apoptosis

**Fig 3:**
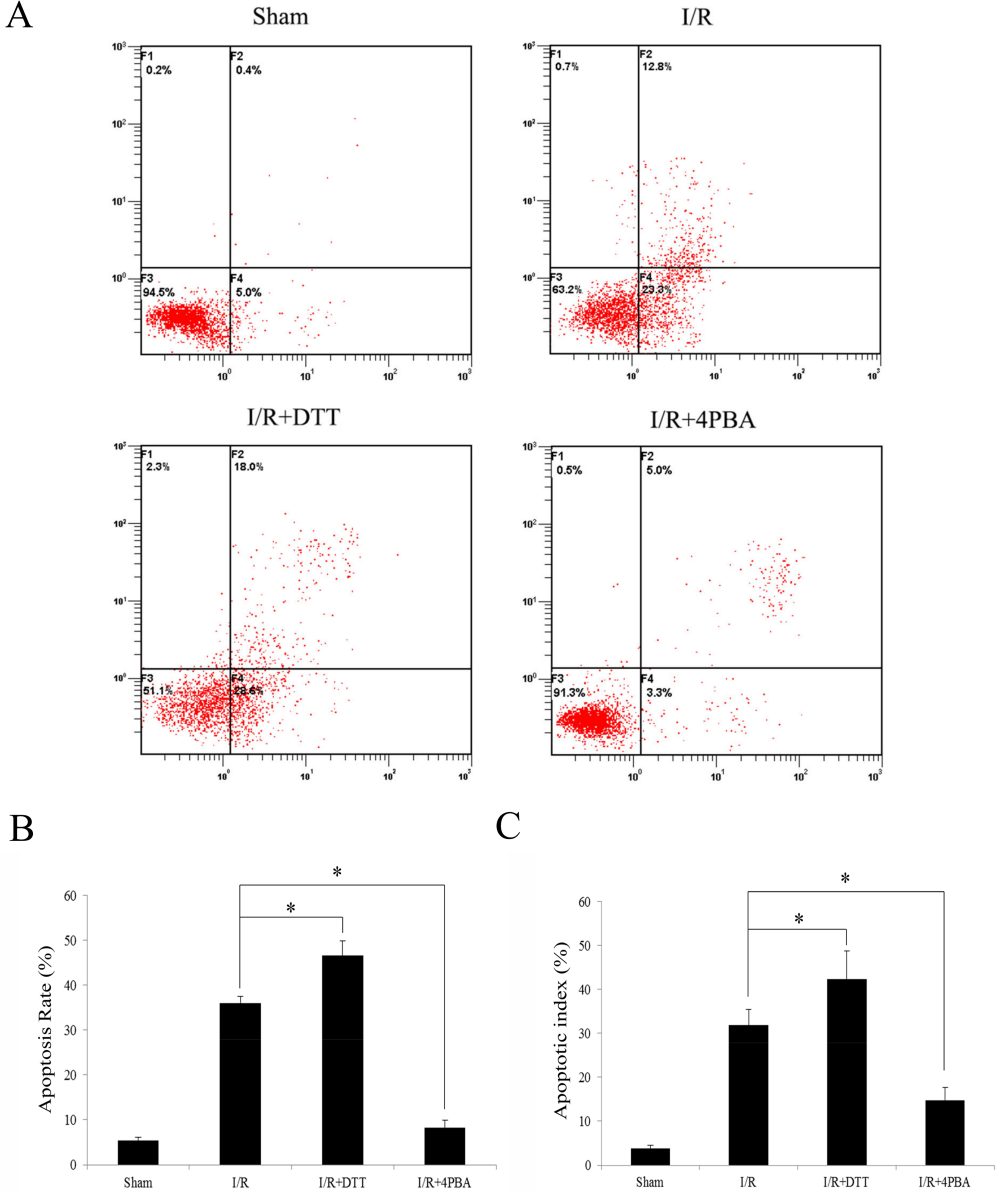
Suppressed UPR attenuate cardiomyocyte apoptosis following myoc myocardial ardium I/R. (A) The results of apoptotic cells with flow cytometry. After the treatment of each group, cells were collected for the detection of cell apoptosis as described previously. (B) The percentage of apoptotic cells was shown after analysis by flow cytometry. (C) Statistical result of apoptotic cells was shown after analysis by the TUNEL assay. Rat hearts were harvested and sectioned for analysis of apoptosis by the TUNEL assay. The data were presented as the mean ± SEM (*P<0.05, n = 6 for each group).

Firstly, we used the Annexin V-FITC and PI staining via flow cytometry to detect the apoptosis of the heart cells in rats with I/R. As shown in Fig 3A and 3B, the ratios of apoptotic cells in I/R+DTT and I/R+4PBA were (46.6±3.3%) and (8.3±0.8%) respectively, compared with I/R group (36.1±1.2%), the apoptosis rate of myocardial cells were significantly increased in I/R+DTT group and significantly decreased in I/R+4PBA group.

The detection of TUNEL positivity is used to evaluate the levels of myocardial apoptosis in rats with I/R. Compared to Sham group, the AIs in the myocardium of rats in the I/R group was significantly increased. Conversely, the AIs in the I/R+4PBA group were significantly lower than those in the I/R group (13.3±2.1% versus 22.33±3.4%, *P*<0.05; Fig 3). The AIs of the I/R+DTT group were higher than the I/R group (*P*<0.05; Fig 3C).

### Cardiac function

**Fig 4:**
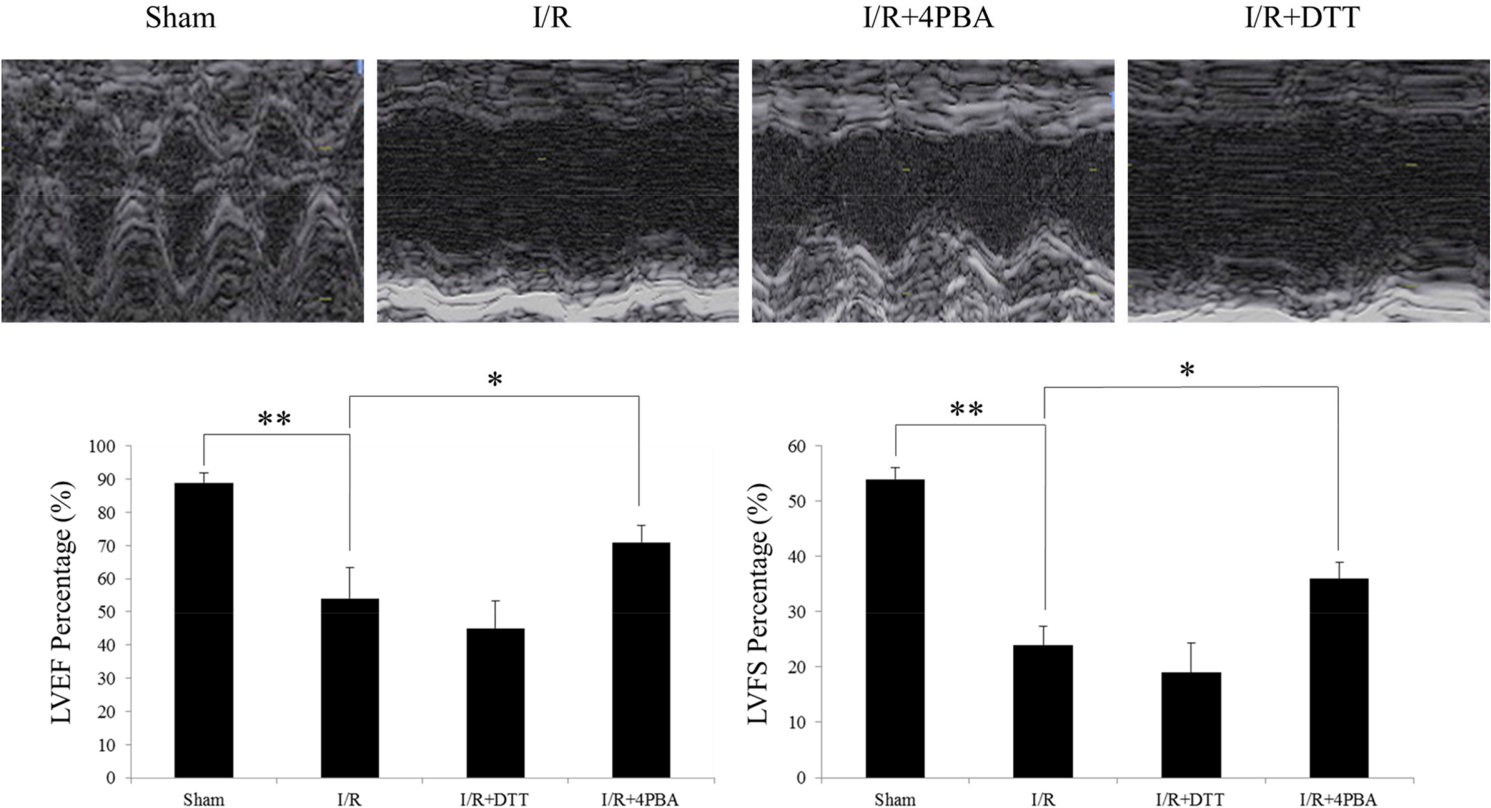
Echocardiography was conducted at 72h after reperfusion. Representative images of echocardiographic examination showed that the 4PBA group has better left ventricular free wall motion compared with the I/R group and DTT group. The data were presented as the mean ± SEM (n = 6 for each group, *P<0.05, **P<0.01).

Echocardiography was conducted at two time points: 10 min before surgery to obtain baseline data and 72 h after reperfusion. No significant difference in the baseline echocardiographic evaluation was observed among these groups (data not shown). I/R injury impaired cardiac systolic function compared with the sham group. For instance, a significant reduction in left ventricular ejection fraction (LVEF; 54±2.1% versus 89±3.3%, *P*<0.05; Fig 4), a decrease in left ventricular fractional shortening (LVFS; 24±2.4% versus 54±4.1%, *P*<0.05; Fig 4). 4PBA treatment restored LVEF after I/R (64±3.6% versus 54±2.1%, *P*<0.05; Fig 4), whereas DTT could aggravate this injury (45±2.8% versus 54±2.1%, *P*<0.05; Fig 4).

### Induction of the molecules of the UPR expression in the Sham group

**Fig 5:**
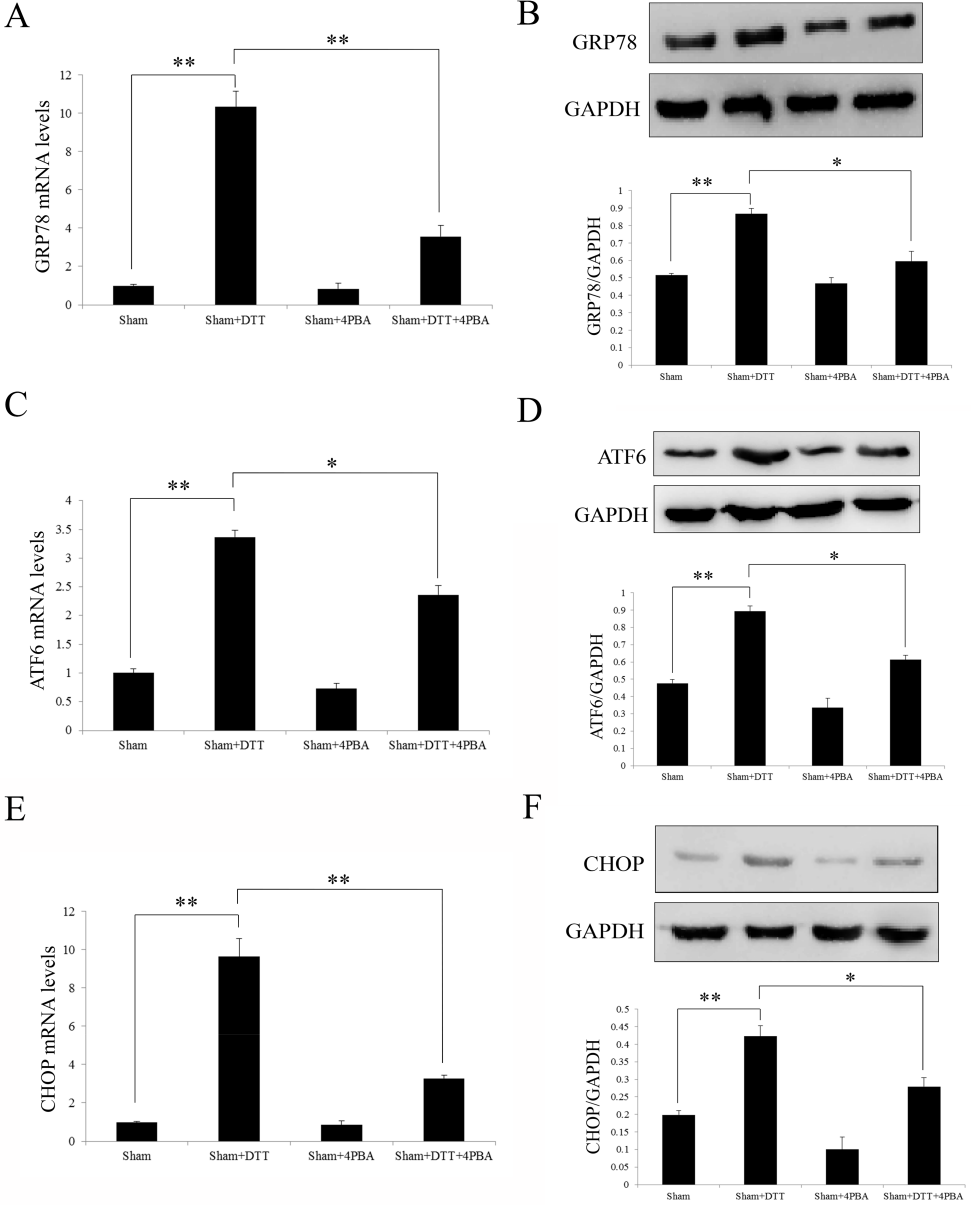
Induction of the molecules of the UPR expression in the Sham group. (A, C and E) Analysis the transcript level of GRP78, ATF6 and CHOP by qRT-PCR. (B, D and F) Representative Western blotting illustrating the expression of GRP78, ATF6, CHOP and GAPDH and the quantitative analysis of them expression level after normalization to GAPDH. The data were presented as the mean ± SEM (Cell lysates from n = 6 hearts/group, *P<0.05, **P<0.01).

Firstly, we affirmed the effect of the SD rats which were pretreated with UPR stimulator DTT and UPR inhibitor 4PBA on UPR signal pathway in the sham group. As shown in the Fig 5, we examined GRP78, ATF6 and CHOP both at transcript and protein levels. The expression of GRP78, ATF6 and CHOP were significantly increased in the Sham+DTT groups(P<0.05; Fig 5), while in the Sham+4PBA group the expressions were not significant changed compared with Sham group. By the way, when pre-treated with both DTT and 4PBA in the Sham group (Sham+DTT+4PAB), the molecules expression of UPR were significantly decreased compared with Sham+DTT group. These results illustrate that the 4PBA can play a suppression effect against to DTT.

### Induction of GRP78 expression after I/R in rats

**Fig 6:**
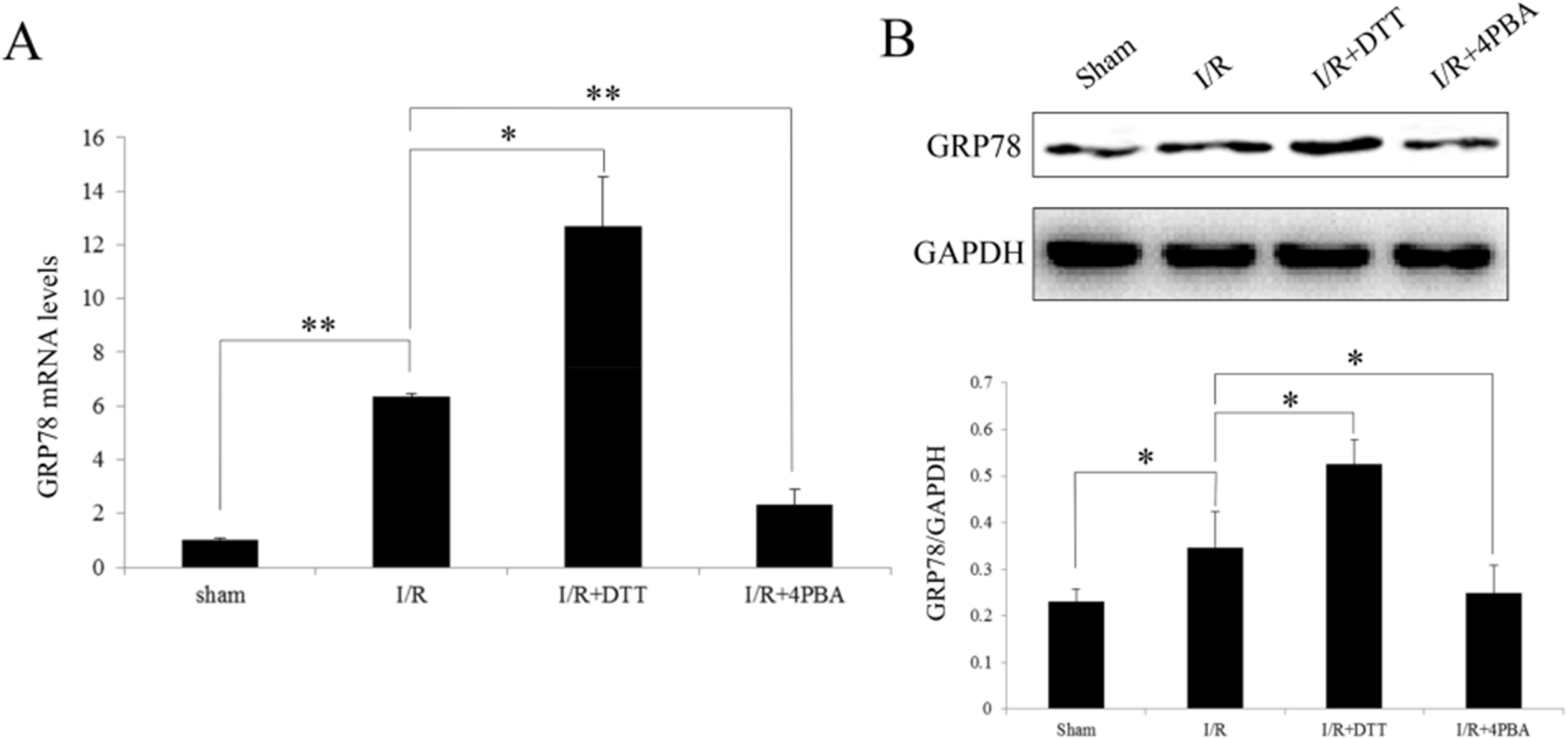
Effect of preconditioning with DTT or 4PBA on the expression of GRP78 after I/R injury in rats. (A) Analysis the transcript level of GRP78 by qRT-PCR. (B) Representative Western blotting illustrating the expression of GRP78 and GAPDH and the quantitative analysis GRP78 expression level after normalization to GAPDH. The data were presented as the mean ± SEM (Cell lysates from n = 6 hearts/group, *P<0.05, **P<0.01).

The GRP78 expression is the hallmark of ER stress since it is activated specifically in the conditions of ER stress. Therefore, in the current study we examined GRP78 at transcript and protein levels after different treatments of I/R injury. Compared to Sham group, the expression of GRP78 was significantly increased in the I/R and I/R+DTT (*P*<0.05; Fig 6), while was not increased in the I/R+4PBA group. Compared to I/R group, GRP78 expression was significantly increased in I/R+DTT group, and in I/R+4PBA group was significantly decreased (*P*<0.05; Fig 6).

### Induction of processed XBP1 after I/R in rats

**Fig 7:**
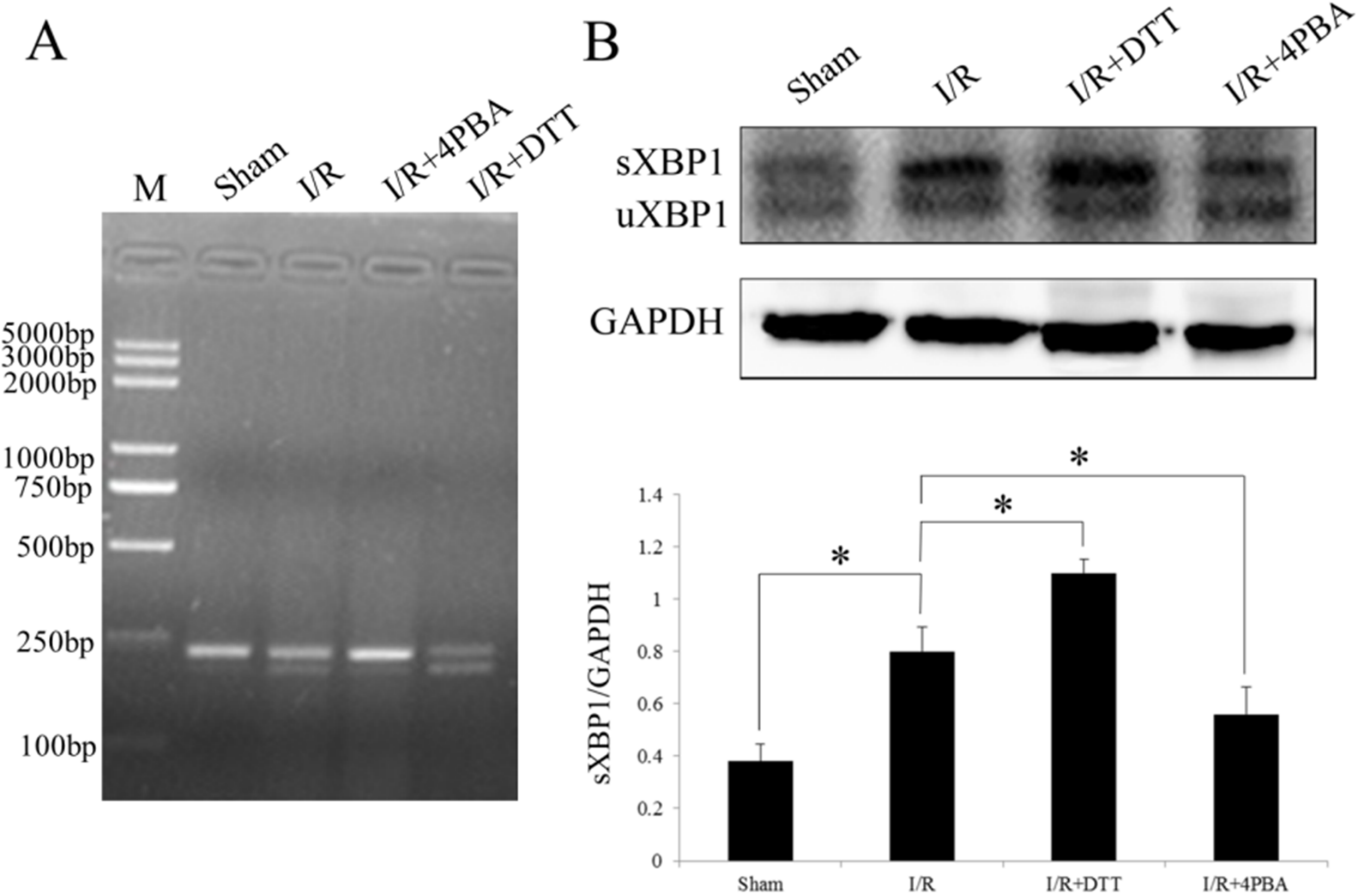
Effect of preconditioning with DTT or 4PBA on the xbp1 processing after I/R injury in rats. (A) Analysis the effect of XBP1 processing by RT-PCR. (B) Representative Western blotting illustrating the expression xbp1 processing and GAPDH and the quantitative analysis spliced XBP1 (sXBP1) expression level after normalization to GAPDH. The data were presented as the mean ± SEM (Cell lysates from n = 6 hearts/group, *P<0.05).

Processed XBP1 mRNA is a reaction that cut out 26 base sequences from XBP1 mRNA, which is implied the activation of IRE1 pathway in unfolded protein response. Therefore, in the current study we examined XBP1 at transcript and protein levels by RT-PCR using specific primers and Western blotting respectively after different treatment of I/R injury. As shown in the Fig 7A, the XBP1 mRNA was obviously processed in the group of I/R and I/R+DTT. Compared to the sham group, the expression of sXBP1 protein was significant increase in the I/R and I/R+DTT group (*P*<0.05; Fig 7B).

### Induction of CHOP expression after I/R in rats

**Fig 8:**
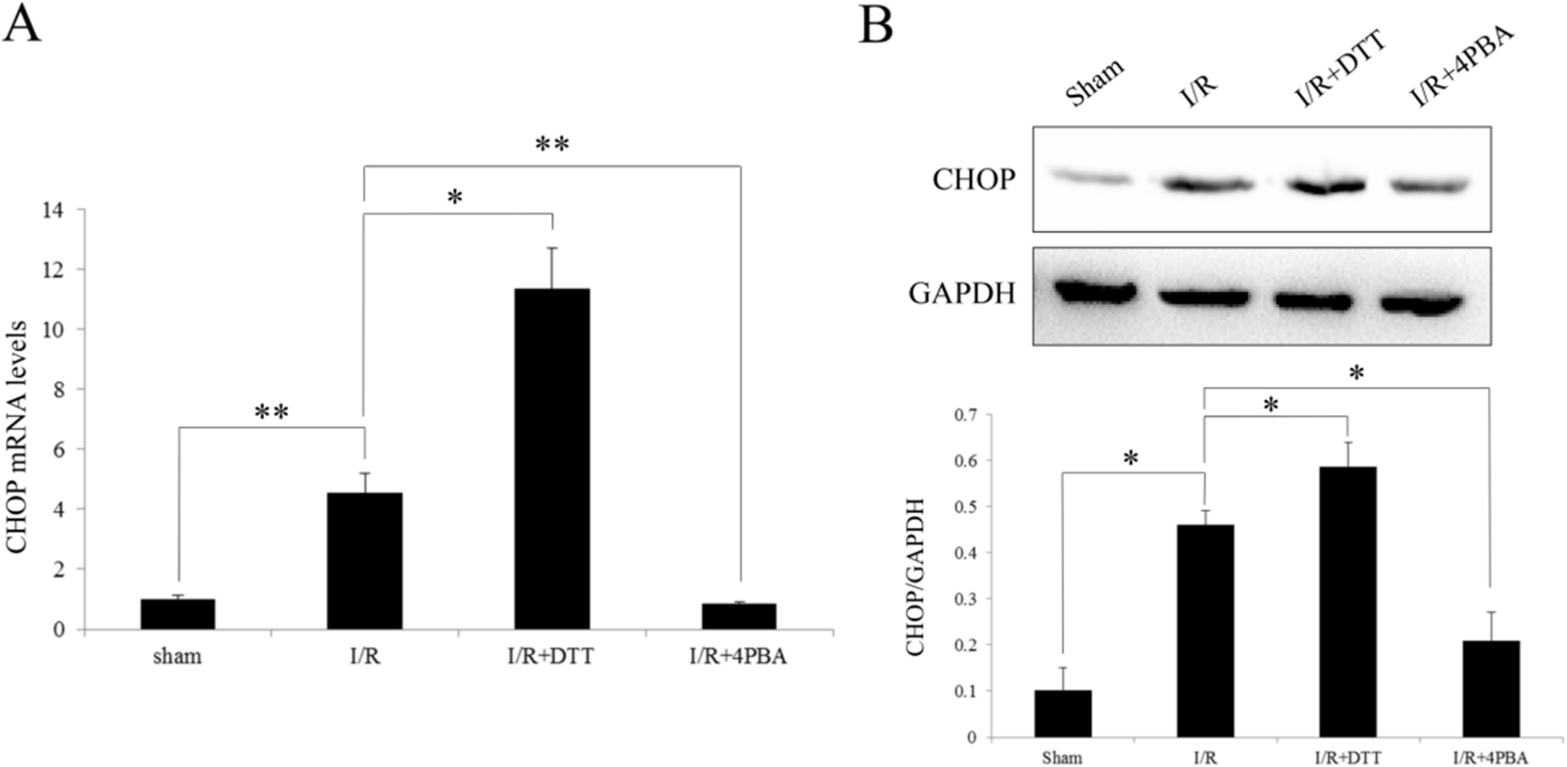
Effect of preconditioning with DTT or 4PBA on the expression of CHOP after I/R injury in rats. (A) Analysis the transcript level of CHOP by qRT-PCR. (B) Representative Western blotting illustrating the expression of CHOP and GAPDH and the quantitative analysis CHOP expression level after normalization to GAPDH. The data were presented as the mean ± SEM (Cell lysates from n = 6 hearts/group, *P<0.05, **P<0.01).

The effects of CHOP up-regulation has been recognized as a sigh of disturb the functioning of the ER. In the I/R and I/R+DTT groups, the CHOP expression was significantly increased both in mRNA and protein levels compared with Sham group (*P*<0.05; Fig 8). Compared to the I/R group, the expression of CHOP in I/R+DTT group was significantly increased, and the I/R+4PBA group is significantly decreased (*P*<0.05; Fig 8). Therefore, those results shown that I/R injury up-regulate the expression of CHOP, which also suggested I/R injury activate the PERK pathway of UPR in rats.

### Induction of ATF6 expression after I/R in rats

**Fig 9:**
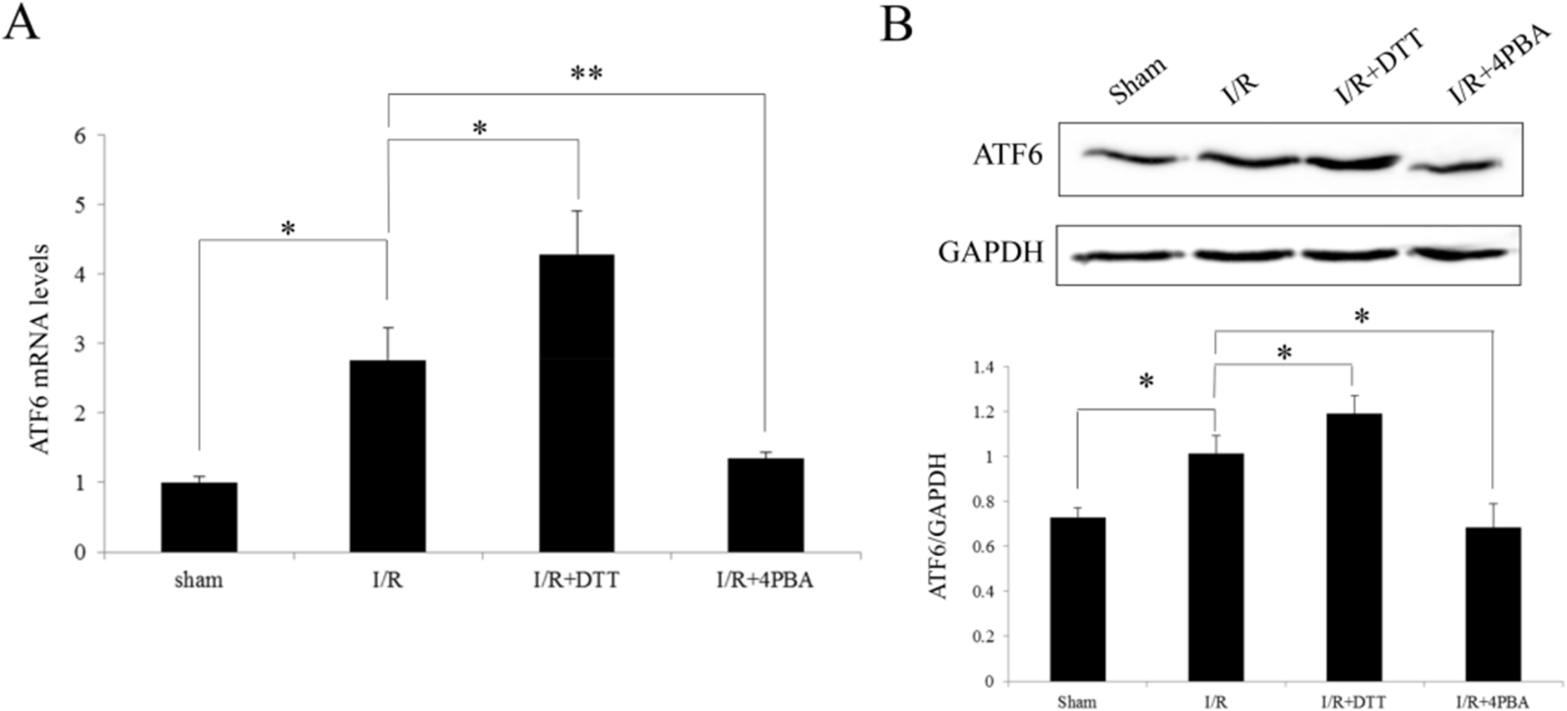
Effect of preconditioning with DTT or 4PBA on the expression of ATF6 after I/R injury in rats. (A) Analysis the transcript level of ATF6 by qRT-PCR. (B) Representative Western blotting illustrating the expression of ATF6 and GAPDH and the quantitative analysis ATF6 expression level after normalization to GAPDH. The data were presented as the mean ± SEM (Cell lysates from n = 6 hearts/group, *P<0.05, **P<0.01).

ATF6 is an upstream of ATF6 pathway, which plays an important role in cell survival mechanisms after ER stress. We detected ATF6 expression both in mRNA and protein levels in the different groups. Compared to Sham group, the expression of ATF6 was significant increase in I/R and I/R+DTT groups (*P*<0.05; Fig 9). Conversely, inhibition of UPR by 4PBA, the expression of ATF6 was decreased in I/R+4PBA group compared with I/R group (*P*<0.05; Fig 9). Taken together, the above results indicated the different expression of ATF6 and CHOP which correlates with the GRP78 expression, the major ER stress marker.

### Induction of caspase-12 and caspase-3 expression after I/R in rats

**Fig 10:**
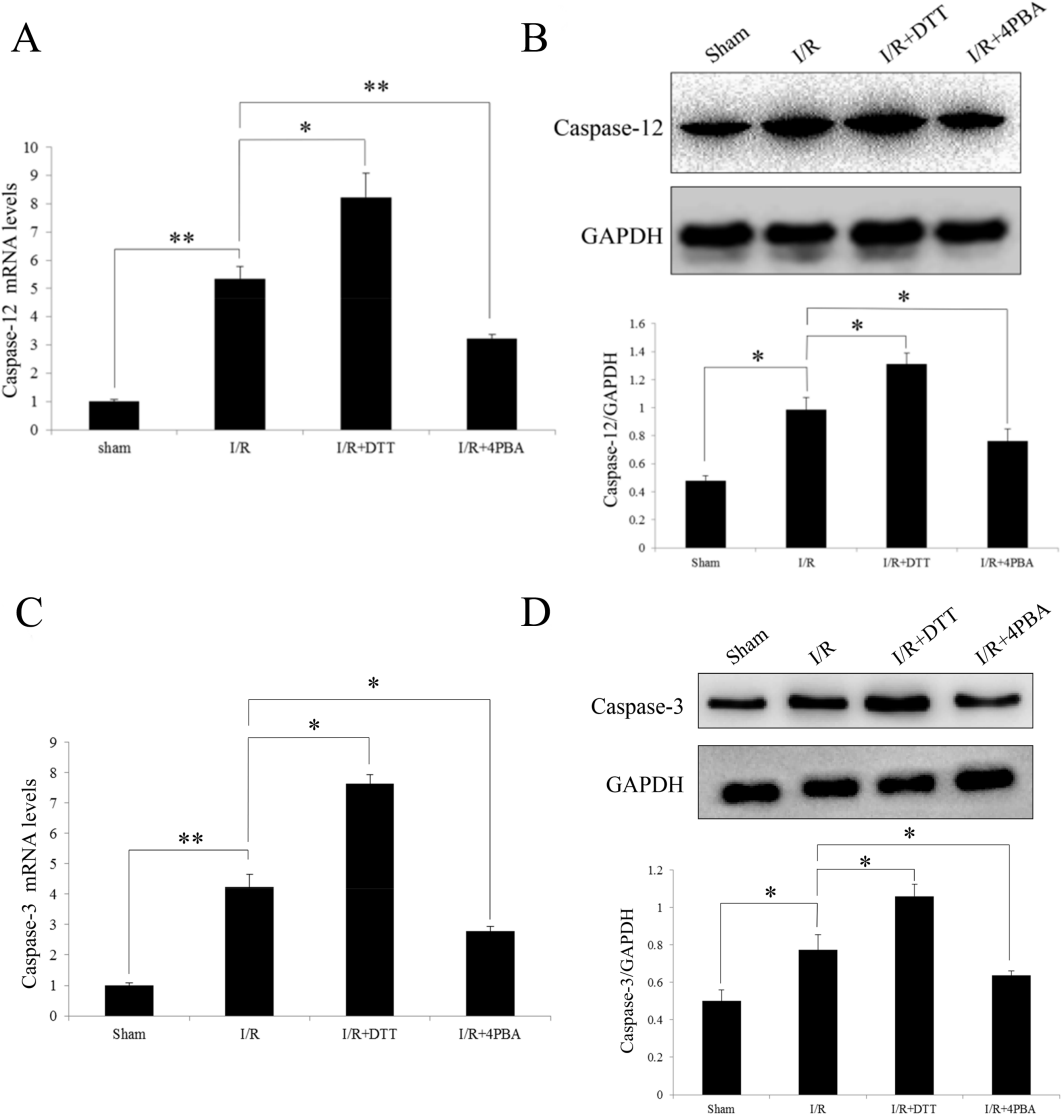
Effect of preconditioning with DTT or 4PBA on the expression of caspase-12 and caspase-3 after I/R injury in rats. (A and C) Analysis the transcript levels of Caspase-12 or Caspse-3 by qRT-PCR. (B and D) Representative Western blotting illustrating the expression of Caspase-12 or Caspase-3 and GAPDH and the quantitative analysis the protein expression level after normalization to GAPDH. The data were presented as the mean ± SEM (Cell lysates from n = 6 hearts/group, *P<0.05, **P<0.01).

After observed the activation of UPR, it was reasonable to detect the expression level of caspase-12 and Caspase-3, which acts a crucial role in ER stress-mediated cell death. Therefore, the transcript and protein levels of caspase-12 or caspase-3 were detected. The results show the expression of caspase-12 was significant increase in I/R and I/R+DTT group compared to the Sham group (*P*<0.05; Fig 10A and 10B). Conversely, when UPR was inhibited, the expression of caspase-12 in the I/R+4PBA group is decreased compared to I/R group (*P*<0.05; Fig 10A and 10B). Corresponding, the expression of the downstream effector Caspase-3 is similar with Caspase-12 (Fig 10C and 10D).

## Discussion

The present study revealed that all the three branches of UPR pathway are involved in the myocardial I/R injury in rats. Furthermore, we also demonstrated that inhibition of UPR reduced myocardium damage after I/R surgery while activation of UPR had opposite effects.

It has been shown that ER stress is associated with many diseases including diabetes, obesity, atherosclerosis, cancer, neurodegenerative diseases and inflammatory diseases [34–36]. Particularly, some heart failures have been shown to associate with enhancement of ER stress, than activate UPR signal pathway [37]. The mechanisms of which ER stress leads to cell death following I/R injury is not elusive. Although multiple potential participants have been described, but little clarity is known about which specific death effectors dominate in particular cellular contexts. In this study, we demonstrated the critical involvement of UPR after I/R injury in rats with the activation of multiple UPR signal pathway regulators. Furthermore, we also demonstrated that inhibition of UPR leads to reduced cardiomyocytes damage after I/R injury.

In the heart, the infarct size and the changed plasma myocardial enzymes are the two characteristics of I/R injury [38]. In the present study, we found myocardium treated with I/R significantly increased infarct size (Fig 1) and plasma CK and LDH activities (Fig 2) in rats. Furthermore, there was more apoptosis cells appeared in DTT treated rat than in the 4PBA treated cells (Fig 3). Pretreatment with 4PBA significantly decreased the infarct size and plasma CK and LDH activities (Fig 2). In the current study, we also showed that preconditioning with 4PBA improved cardiac functionality in rats with myocardial I/R injury (Fig 4). All these results indicate that suppress cell UPR has a cardioprotective effect against myocardial I/R injury.

Under ER stress condition, unfolded or misfolded proteins accumulate in the ER lumen, a pathologic process resulting in the activation of the UPR pathway to combat the harmful effects of ER stress. However, little is known about the exact mechanism of UPR signaling pathways after myocardial I/R injury in rats. Therefore, we examined the signal molecules of three branches of UPR to clarify which pathway is activated.

Increased expression of GRP78 serves as a hallmark of ER stress. An increased expression of GRP78 both in mRNA and protein levels were found in the cardiomyocyte indicated I/R injury induced ER stress (Fig 6). In the physiological state, GRP78 binds to the three effectors of UPR (ATF6, PERK and IRE1) to suppress their activity [39]. Under ER stress condition, when misfolded proteins accumulate in the ER lumen, GRP78 dissociates from these effectors allowing their activation. Upon activation, the endonuclease activity of IRE1 specifically cuts out a 26-nucleotide intron from the xbp1 mRNA, which leads to a shift of the open reading frame of xbp1 mRNA [15]. Therefore, processing of xbp1 mRNA is an indication of activation UPR branch IRE1 pathway and ER dysfunction. In the present study, we indeed observed a increasing of the processed xbp1 mRNA and protein level after I/R surgery or treated with UPR stimulator DTT (Fig 7). Processed xbp1 mRNA is translated into a new protein of 54 kDa that functions as a transcription factor and has diverse targets specificity for ER stress genes including GRP78 [40]. Thus, IRE1–xbp1 pathway seems to be pro-survival by activating the transcription of ER chaperones. However, prolonged ER stress accompanied by failure of adaptive response may eventually results in apoptotic cell death.

There are many studies verified that CHOP participates in apoptosis signaling pathways and the increasing expression of CHOP mRNA serves as a symbol of the PERK signal pathway activation [13, 41]. Activated PERK phosphorylates eIF2a to avoid further accumulation of proteins by suppressing protein synthesis but also leads to the paradoxically increased expression of transcription factors ATF-4 and CHOP [13]. The increased mRNA and protein level of CHOP observed in the present study after I/R injury or treated with DTT(Fig 8) are also in accordance with the previous study after bilateral common carotid artery occlusion in mice [42]. Thus, it indicates that CHOP might play a crucial role in mediating the ER stress-induced apoptotic cell death pathways after ischemic injury. The temporal profile of ATF6 (Fig 9) and CHOP, GRP78 and processed xbp1 mRNA collectively demonstrated the activation of UPR three signaling pathways, ATF6, PERK and IRE1 after I/R injury.

Caspase-12, located on the ER, is a molecule that recognized as a sign in cell death pathway especially related to ER stress [27, 43]. During ER stress, caspase-12 dissociates from the ER membrane and then to be activated and initiates downstream apoptotic pathways. Compared to sham group, we have observed that the expression of caspase-12 was significant increase in the I/R and DTT group (Fig 10A and 10B). Caspase-12 deficient mice are also resistant to ER stress-induced apoptosis [44]. Corresponding, the expression of the downstream effector Caspase-3 is similar with Caspase-12 (Fig 10C and 10D). Interestingly, in this study, we observed the increasing of caspase-12 and caspase-3 appeared to correlate with that UPR molecules of GRP78, ATF-6, and CHOP expression observed in the present study. Our results showed that the activation of caspase-12 and caspase-3 were significantly suppressed by 4PBA.

A number of experimental reperfusion therapies have been shown to ameliorate myocardial reperfusion injury, although these beneficial effects have barely been translated into clinical practice. One significant reason for this disparity is thought to be the difficulty of delivering drugs to the right component at the right time and dosage in myocardial I/R.

In summary, this study has provided a new concept of how UPR play an important role in myocardial I/R injury in rats, which is associated with CHOP-Caspase12 apoptosis pathway, and this may be one of promising therapeutic targets for I/R injury treated.
